# Discovery of Five New Ethylene-Forming Enzymes for Clean Production of Ethylene in *E. coli*

**DOI:** 10.3390/ijms23094500

**Published:** 2022-04-19

**Authors:** Yixuan Cui, Ying Jiang, Meng Xiao, Muhammad Zeeshan Munir, Sadaf Riaz, Faiz Rasul, Maurycy Daroch

**Affiliations:** School of Environment and Energy, Peking University Shenzhen Graduate School, 2199 Lishui Rd., Shenzhen 518055, China; 1901213194@pku.edu.cn (Y.C.); jiangy@stu.pku.edu.cn (Y.J.); 1901213206@pku.edu.cn (M.X.); jiger007@pku.edu.cn (M.Z.M.); sadaf.riaz@pku.edu.cn (S.R.); frasul@pku.edu.cn (F.R.)

**Keywords:** ethylene forming enzyme, ethylene, *E. coli*, protein expression, TCA cycle, alpha-ketoglutarate

## Abstract

Ethylene is an essential platform chemical with a conjugated double bond, which can produce many secondary chemical products through copolymerisation. At present, ethylene production is mainly from petroleum fractionation and cracking, which are unsustainable in the long term, and harmful to our environment. Therefore, a hot research field is seeking a cleaner method for ethylene production. Based on the model ethylene-forming enzyme (Efe) AAD16440.1 (6vp4.1.A) from *Pseudomonas syringae pv. phaseolicol*, we evaluated five putative Efe protein sequences using the data derived from phylogenetic analyses and the conservation of their catalytic structures. Then, pBAD expression frameworks were constructed, and relevant enzymes were expressed in *E. coli* BL21. Finally, enzymatic activity in vitro and in vivo was detected to demonstrate their catalytic activity. Our results show that the activity in vitro measured by the conversion of α-ketoglutarate was from 0.21–0.72 μmol ethylene/mg/min, which varied across the temperatures. In cells, the activity of the new Efes was 12.28–147.43 μmol/gDCW/h (DCW, dry cellular weight). Both results prove that all the five putative Efes could produce ethylene.

## 1. Introduction

Ethylene is the simplest olefin. It can be copolymerised into polyethylene [1], ethylene-propylene rubber [2], ethylene acrylic acid [3], and other polymeric materials. These materials are widely used as the building blocks for essential consumer products such as plastics, cosmetics, and paints. It can also be used to produce organic synthetic raw materials like ethanol, acetaldehyde, ethylene chloride, and ethane bromide through organic synthesis or halogenation reactions. Additionally, it can be reacted with methane to catalyse the synthesis of C5–C10 gasoline-grade hydrocarbons [4]. In 2019, world demand for ethylene production reached 170 million tons, and ethylene production rose to 190 million tons per year. Increasing demand and production of ethylene have brought economic growth, but environmental problems remain.

As one of the leading chemical products, ethylene is mainly obtained through petroleum fractionation and hydrocarbon cracking [5]. However, the feedstock, crude oil, is non-renewable and ultimately unsustainable. Therefore, recent research mainly focuses on reducing pollution by improving the chemical production process [6] or developing a cleaner method through biological processes [7]. The biosynthetic approach is auspicious and results in no other pollutants being produced. Besides, it is easier to separate ethylene from the cell culture environment than other hydrocarbons because of its volatility and insolubility, making biosynthetic ethylene production even more appealing.

**Figure 1:**
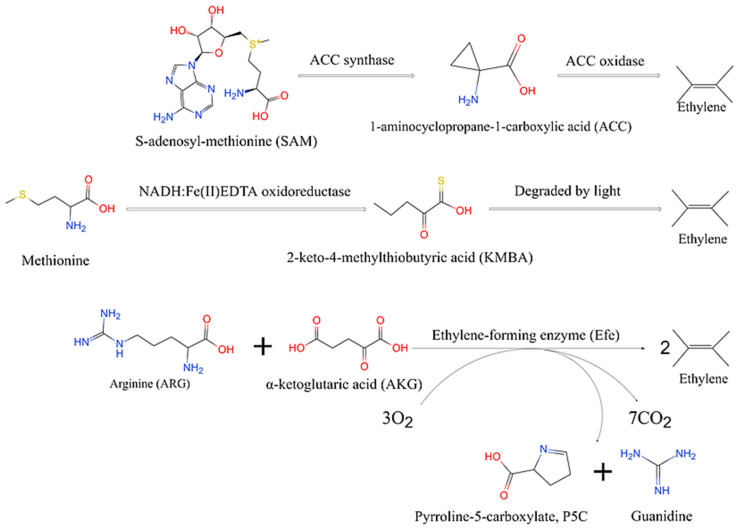
Biosynthetic pathways of ethylene synthesis.

Up to now, three pathways of ethylene biosynthesis have been elucidated (Figure 1). In higher plants, such as tomatoes, S-adenosyl-methionine (SAM) is converted to 1-aminocyclopropane-1-carboxylic acid (ACC) with the help of ACC synthase and then converted to ethylene by ACC oxidase [8,9]. In these plants, ethylene works as a plant hormone and as a defence response to biotic and abiotic stresses, like pathogen attacks [10]. Based on that, some plant-associated microorganisms have shown ethylene-production ability [11]. In microorganisms, there are two pathways for ethylene production. The first of these pathways is present in bacteria and fungus (*Filobasidiaceae*). The biosynthetic cascade commences with NADH:Fe(III)EDTA oxidoreductase that converts into 2-keto-4-methylthiobutyric acid (KMBA), which is subsequently degraded to ethylene by light [12,13]. This process is affected by both carbon and nitrogen sources [14]. In the second pathway, α-ketoglutarate (AKG) and L-arginine (ARG) are the primary substrates to produce ethylene [15]. The ethylene-forming enzyme (Efe) catalyses the reaction in which the ratio of precursor and product was 1:1:2 [7]. In *E. coli* and some other industrial strains, the substrate for this reaction, AKG, is mainly produced by the microorganism’s tricarboxylic acid cycle (TCA cycle) [16,17] without an additional precursor requirement.

Efe is an important enzyme characteristic for the second microbial pathway mentioned above. It is a 2-oxoglutarate (2OG)-dependent nonheme iron(II) oxygenase (2OG is AKG), which catalyses the oxidation-decarboxylation of AKG to succinate and CO_2_. In the process of ethylene synthesis by Efe [18], the Cδ atom of ARG can be hydroxylated by Efe to form intermediate products, which are rapidly decomposed into guanidine and 1-pyrroline-5-carboxylate (P5C). Meanwhile, O_2_ is activated at the same active site, converting AKG into ethylene and three molecules of CO_2_ [19,20]. The 2-fold catalytic reaction of Efe demonstrates the remarkable flexibility of the active site of this enzyme, which provides the means to perform these two unique O_2_ activation reactions. At the same time, the study showed [21] that the addition of iron(II), arginine, and AKG improved the stability of the enzyme, and the combination of substrate and cofactor had a synergistic effect. Efe was first reported in *Pseudomonas solanacearum* [22]. The two best-described enzymes that catalyse this reaction are from *P. syringae pv. phaseolicola* (Kudzu strain) and *P. syringae pv. glycinea* [11]. Now, the ethylene-forming enzyme gene (*efe* gene) from *P. syringae* has been successfully expressed in *E. coli* [23,24], *Pseudomonas putida* [25], *Saccharomyces cerevisiae* [26], *Trichoderma reesei* [27], and in different strains of cyanobacteria [28,29,30]. The activity of ethylene production is 0.093 μmol/gDCW/h in *Trichoderma viride* [31] to 2859.2 μmol/gDCW/h (gDCW is dry cellular weight) in *Pseudomonas putida* [32]. The production rate in *E. coli* ranges from 10.9 μmol/gDCW/h in DH5α (RS101 ori) [33] to 625 μmol/gDCW/h in DH5α (pUC18 ori) [23].

In this work, five putative Efe sequences from different species of cyanobacteria and proteobacteria were selected using the structural and sequence information of a model Efe sequence from *P. syringae pv. phaseolicola* as a reference. The structures of these biocatalysts were predicted with a homology-based approach. On this base, we report the heterologous expression of the six Efes in *E. coli* using a low-copy, arabinose-inducible vector, pBAD_LIC_cloning vector (8A), analysed their activity in vitro under different temperatures, and calculated the productivity of ethylene in *E. coli* cells. 

## 2. Results

### 2.1. Sequences Discovery and Phylogenetic Analysis

**Figure 2:**
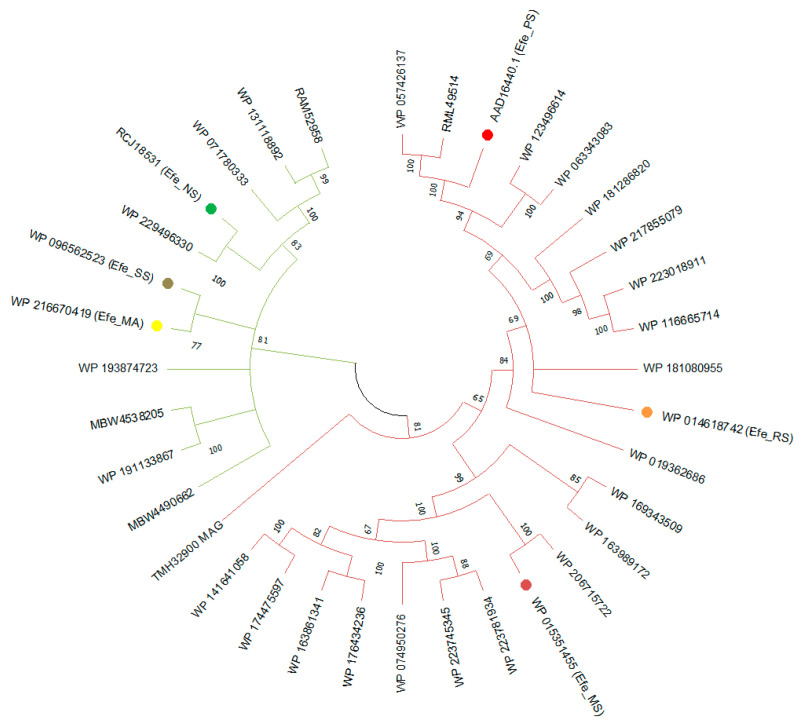
ML-Boostrap-phylogenetic tree of Efe-homologous sequences. The green branches of the tree indicate cyanobacteria and rose-red proteobacteria. The sequences used in the next step are marked with dots of relevant different colours.

Thirty-four amino acid sequences belonging to cyanobacteria (green branches) and proteobacteria (rose red branches) with significant sequence similarities and fold recognition to the Efe sequence AAD16440.1 (marked with a red dot) were selected. The relationship between these strains was inferred using the phylogenetic tree (Figure 2, Table S1). 

Most of them were initially described as isopenicilin N synthase family oxygenase by the automatic annotation systems, while the others were MFS transporter, 2OG-Fe (II) oxygenase, or some other uncertain proteins. As representatives of the branches and guided with fold recognition data, we selected five sequences from different strains (marked with points): Yellow point: *Microcoleus asticus* (GenBank: NQE34890, RefSeq: WP_216670419.1), Efe_MA; Rose red point: *Myxococcus stipitatus* DSM 14675 (RefSeq: WP_015351455.1, source: CP004025), Efe_MS; Orange point: *Ralstonia solanacearum* (RefSeq: WP_014618742.1, source: CP012944), Efe_RS; Sumdge point: *Scytonema* sp. NIES-4073 (RefSeq: WP_096562523.1), Efe_SS; Green point: *Nostoc* sp. ATCC 43529 (GenBank: RCJ18531, RefSeq: WP_019362686.1), Efe_NS, for further analysis to verify their ethylene-forming activity.

### 2.2. Structural Modelling and Analysis

**Figure 3:**
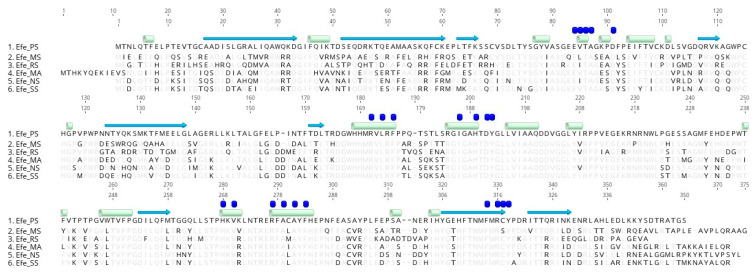
Amino acid sequence alignment (α-helix: green box, β-strand: blue arrow, activity sites: blue box) between five proteins and AAD16440.1 (Efe_PS).

Sequence alignment and structural modelling were performed with AAD16440.1 (PDBID: 6vp4.1.A) (Figure 3, Figure S1). SWISS-MODEL shows that these models have a high matching degree. Their parameters like Seq Identity, GMQE, QMEANDisCo Globa, and QMEAN indicated model reliability (Table S2).

The comparison between the five proteins and the reference structure showed 9 groups of α-helix structures and 18 groups of β-strand structures in the model. There were some differences in the sequences of α-helix structures and β-strands structures based on the sequence alignment (Figure 3). Structural alignment using SWISS-MODEL (Figure S1a) predicted that Efe_MS does not change in α-helical or β-strand structures. Efe_RS has a more extended β-strand structure in R.67 (6vp4.1.A: S.67) and one extra β-strand structure in M.157, E.158, and T.159 (6vp4.1.A: N.157, T.158). Efe_MA has a more extended β-strand structure in S.77 and Q.68 (6vp4.1.A: S.67 and S.68) and the β-strand structure in T.160, D.161, and L.162 in 6vp4.1.A change into N.168, A.169, L.170, and T.171 there. In Efe_NS, the β-strand structure located in T.160, D.161, and L.162 in 6vp4.1.A also change into D.158, A.159, L.160, and T.161. Efe_SS does not have any change in α-helix or β-strand structures. All above changes are minor for the location of β-strand or the formation of a short helix, indicating structural conservation of these enzymes. They may not influence the based structure or work as an Efe. What is more, structural alignment results using PyMOL shows a minor difference in the structure (Figure S1c). The 6VP4.1.A model showed that Efe mainly contained three ligand sites (Figure S1b), including 1 × ARG, 1 × AKG, and 1 × FE binding site, which were located in: E.84, V.85, T.86, A.87, D.91, R.171, L.173, F.175, I.186, H.189, D.191, Y.192 L.206, H.268, V.270, R.277, A.279, A.281, F.283, F.314, R.316, C.317, and Y.318. The results also showed that all the five proteins were conserved at the binding sites of Fe and AKG, while at the ARG binding site V.85 (Efe_MS: L.85, Efe_RS: I.85, Efe_NS: I.85) and C.317 (Efe_MS: S.317) were found unconserved. V.85 is located at a residue and at hydrogen bond sites, which is relatively important. But V, I, and L are all non-polar amino acids containing fatty hydrocarbon side chains, which means this change is unlikely to have a major effect on the structure and function of the ARG ligand site. Therefore, we preliminarily predicted that the change of V.85 into L.85 or I.85 has little impact on ARG-binding and enzymatic activity. Besides, C.317 is at another residue, and according to the analysis of disulfide bond formation of a cysteine residue in the template model 6VP4.1.A (shown in Figure S2), the distance of C.317 from other cysteine sites is more than 10A, which is too far to form disulfide bonds. Therefore, we preliminarily speculated that the change from C to S at this site in efe_MS might not be significant and affect its binding to ARG.

### 2.3. Expression of Ethylene Forming Enzymes in E. coli BL21

Six pBAD-*efe*^+^ expression frameworks (Figure S3) were constructed using the homology-based method in *E. coli* DH5α and transformed to BL21 strain for expression. To trace the growth of each *E. coli* BL21_*efe^+^* strain in LB medium, supplemented with arabinose and antibiotic (ampicillin) at different temperatures, the OD_600,_ and pH of the culture were measured.

As is shown in Figure S4, low temperature (20 °C) slightly inhibited the growth rate of *E. coli*, but the effect was not significant: the number of cells still increased and could grow to a high enough concentration. Meanwhile, pH in the culture was stable at 6.9–7.3, which reproves arabinose has little effect on the cultivation system, and metabolic flux was not directed towards acid production but presumably directed towards the TCA cycle as required for the Efe activity. Besides, compared the growth of BL21_*efe^+^* to wild type BL21 (WT), the growth curves were similar, indicating that the insertion of the Efe expression framework had no specific effect on the growth of *E. coli* cells.

### 2.4. Improvement of the Induction Environment

RT-qPCR and SDS-PAGE were used to analyse the arabinose induction effect using BL21_*efe_PS^+^* as the representative strain. In cultivation system, different concentrations of arabinose (0, 0.02%, 0.2%, 2%) and different temperatures (20 °C, 30 °C) were set as the variate.

**Figure 4:**
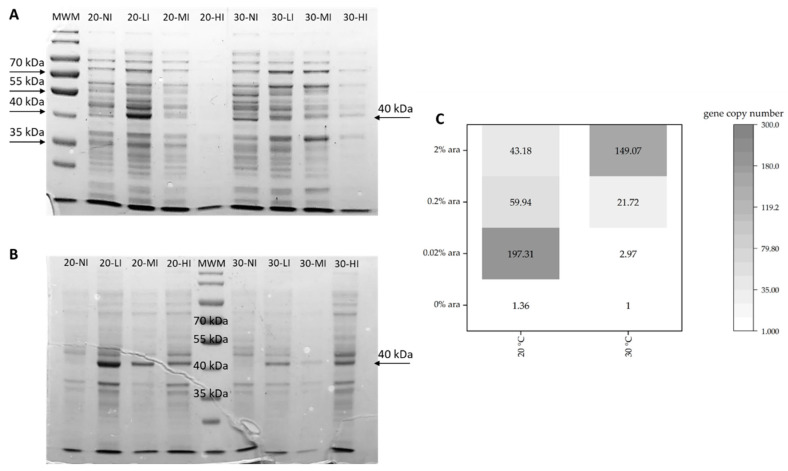
SDS-PAGE (**A**,**B**) and RT-qPCR (**C**) analysis in different level of arabinose and temperatures (BL21_*efe_PS^+^* as a representative). (**A**) Crude extract in supernatant; (**B**) Crude extract in insoluble fraction. The temperature, arabinose concentration and proportion of target protein in each channel of supernatant is: MWM: protein ladder (10–180 kDa, Thermofisher, Waltham, MA, USA); 20-NI: 20 °C, 0% ara: 8.2% proportion; 20-LI: 20 °C, 0.02% ara: 13.5% proportion; 20-MI: 20 °C, 0.2% ara: 8.8% proportion; 20-HI: 20 °C, 2% ara: 6.1% proportion; 30-NI: 30 °C, 0% ara: 8.9% proportion; 30-LI: 30 °C, 0.02% ara: 10.6% proportion; 30-MI: 30 °C, 0.2% ara: 9.1% proportion; 30-HI: 30 °C, 2% ara: 10.3% proportion.

According to the relative gene copy number of BL21_*efe_PS^+^* from RT-qPCR and protein expression from SDS-PAGE in different conditions (Figure 4), the gene was expressed in all the conditions. It was best expressed (mRNA gene relative copies: 197.31, the proportion of target protein of crude extract in the supernatant was approximated as 13.5% predicted by ImageJ) with 0.02% concentration of arabinose at 20 °C. The temperature of 30 °C and high arabinose concentration resulted in the formation of insoluble inclusion bodies that had a negative effect on the performance of the engineered strains. Additional challenges may be associated with an excessive draw of TCA cycle intermediates at high enzymatic that compromise the growth rate at high enzymatic activities of the ethylene-forming enzyme. Therefore a careful balance of temperature, inducer concentration, and induction time is needed to maximise productivity. Therefore, a relatively modest temperature of 20 °C and a 0.02% concentration of arabinose were used in the next step of this work.

### 2.5. Heterologous Expression of Each BL21_efe^+^ Strain

In this experiment, six BL21_*efe^+^* strains were cultured in LB with 50 μg/mL ampicillin and 0.02% arabinose at 20 °C for 5 h.

**Figure 5:**
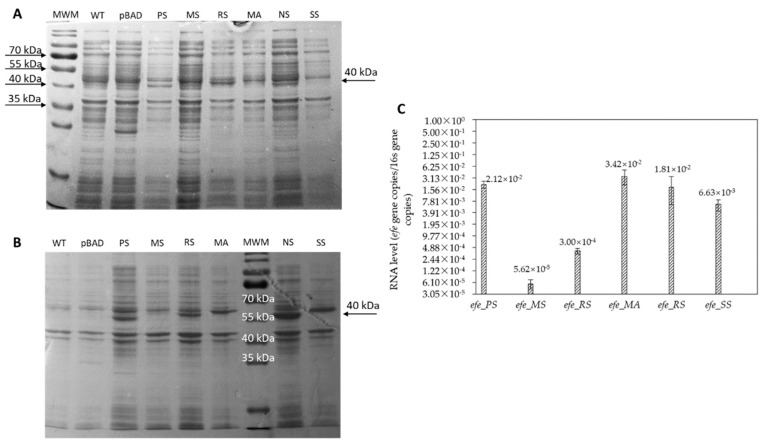
Heterologous expression of the six Efe strains. (**A**) SDS-PAGE of crude extract in supernatant, (**B**) SDS-PAGE of crude extract in insoluble fraction: MWM: protein ladder; WT: BL21 wild type; pBAD: BL21 with pBAD vector; PS: Efe_PS, 39.8 kDa, 12.7% proportion of target protein of crude extract supernatant; MS: Efe_MS, 41.0 kDa, take 5.6% proportion; RS: Efe_RS, 39.6 kDa, take 7.5% proportion; MA: Efe_MA, 42.5 kDa, take 8.9% proportion; NS: Efe_NS, 41.8 kDa, take 7.1% proportion; SS: Efe_SS, 39.2 kDa, take 8.0% proportion. (**C**) RT-qPCR of *efe* genes in the six strains: it is calculated by the ratio of *efe* gene copies to 16sRNA gene copies.

RT-qPCR (Figure 5C) was used to test the mRNA level. First, the standard curves were drawn to calculate the absolute gene copy numbers. PCR efficiency of these curves was around 90–110% (Figure S5), proving that primer and standard curves were accurate. Then, the absolute gene copy number of *efe* genes was compared to the absolute gene copy number of the 16sRNA gene to get the relative gene copy number shown in Figure 5.

SDS-PAGE (Figure 5A,B) was used to analyse the protein production in the crude extract. The bands of Efe protein were around 39–42 kDa. Their identity was also confirmed by a protein MS Q-E test (Table S3, Figure S6). Analysis of the insoluble fraction using SDS-PAGE showed that the proportion of target protein of crude extract in the supernatant was low (5.6–12.7%), and some inclusion bodies were also formed. It may be because of the excessive protein concentration or challenges in the protein folding process. It may influence the productivity of ethylene and need to be improved. 

**Figure 6:**
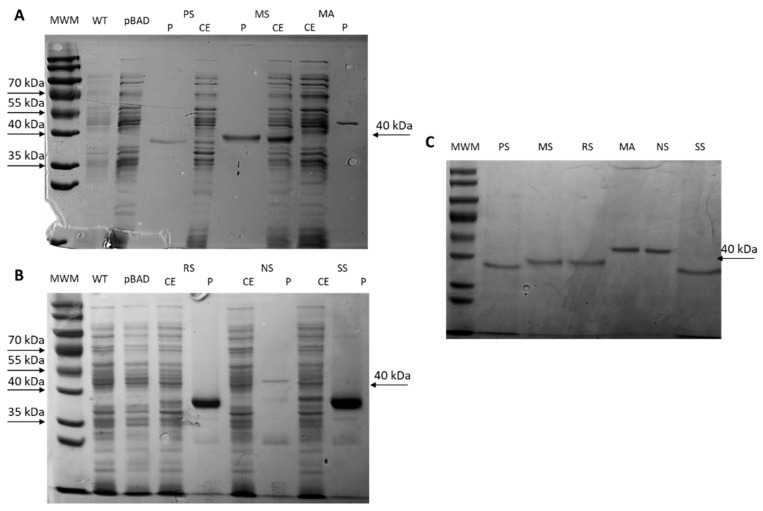
Purification and SDS-PAGE quality control of six expressed Efes; CE: unpurified crude extract, P: purified protein; WT: BL21 wild type; pBAD: BL21 with pBAD vector. (**A**) Expression and purification of Efe_PS, Efe_MS, and Efe_MA constructs; (**B**) Expression and purification of Efe_RS, Efe_NS, and Efe_SS constructs; (**C**) Quality control showing equal loadings of the six purified proteins.

Efes with his-tag were purified with Ni beads (Figure 6). Protein concentration measured by BCA kit was: Efe_PS: 4.3 μg/μL; Efe_RS: 1.107 μg/μL; Efe_MS: 8.88 μg/μL; Efe_MA: 5.38 μg/μL; Efe_NS: 3.97 μg/μL; Efe_SS: 5.55 μg/μL. Then, they were adjusted to the same protein concentration and used for the activity test in the next step (Figure 6C).

### 2.6. Enzymatic Activity In Vitro

Enzymatic activity in vitro was calculated by converting AKG to ethylene in the reaction system. The K_m_ and k_cat_ of each Efes were calculated and shown in Table 1. The K_m_ of the six Efes are 15.1–45.9 μM and 19.8–37.3 μM for AKG and ARG, respectively. In most of Efes, the K_m_ of ARG and AKG is similar, which means they have a fair affinity to these Efes. This is concurrent with previous studies where K_m_ (ARG) was determined as 18 μM and K_m_ (AKG) as 19 μM [15]. However, Efe_MA and Efe_SS have a higher K_m_ (ARG) than K_m_ (AKG), which means a higher concentration of ARG in the reaction system may be more important for the reaction speed or vice versa.

**Figure 7:**
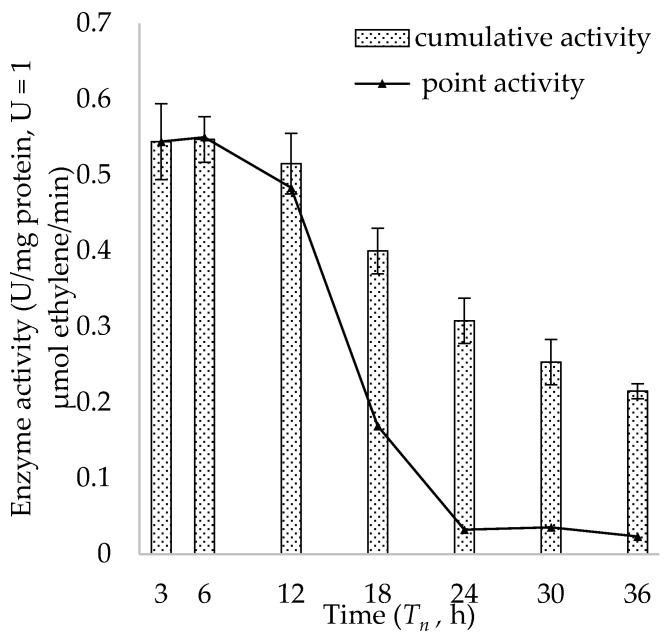
The change of enzymatic activity in vitro (Efe_PS as a representative) with time at 20 °C: cumulative activity: activity from 0 h to T_n_ h; point activity: activity from T_n__−1_ h to T_n_ h.

Efe_PS was used to explore the change of enzymatic activity in vitro with time at 20 °C. Samples were drawn from the reaction system at different time points (T_n_) to detect the activity. The cumulative activity (activity from 0 h to T_n_ h) and the point activity (activity from T_n−1_ h to T_n_ h) in vitro are both calculated (Figure 7). The activity remained high before 12 h dropped subsequently, and the half-life (t_1/2_) of the enzyme was around 12–18 h.

**Figure 8:**
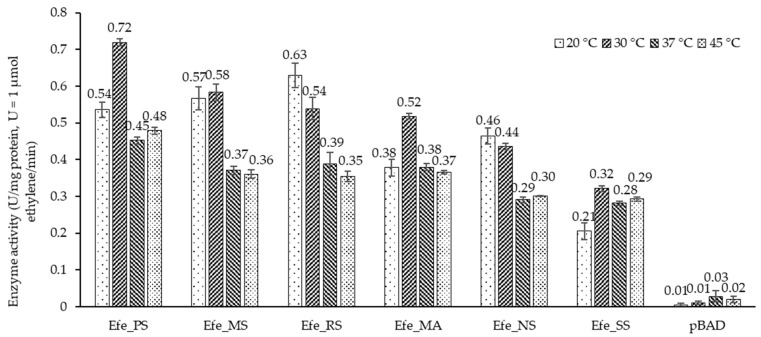
Ethylene production activity in vitro for 12 h at different temperature intervals from 20 °C to 45 °C (calculated by the conversion rate of AKG, 1AKG + 1ARG → 2 Ethylene).

Based on these results, 12 h was set as the reaction time for the temperature study of activity in vitro. As is shown in Figure 8, all the six Efes had a higher activity at the temperature of 20 °C or 30 °C. Although they still were capable of catalysis at 37 °C and 45 °C, their conversion rates were approximately half of the highest reported. Besides, there was little difference between the catalytic activity of the six enzymes. All the purified proteins worked well in vitro and proved that the minor alterations of the ARG ligand site in the structure do not significantly affect ARG binding. This result is concurrent with bioinformatic analyses. During the purification process, the enzymes were purified to homogeneity (Table 2), but the purification yield was relatively low, ranging from 20–40%.

### 2.7. Ethylene Production in Cells

According to the selection of induction conditions and the study of enzymatic activity in vitro, enzymes were well expressed and kept active under 0.02% arabinose in LB at 20 °C. Therefore, these parameters were set as the ethylene production condition.

**Figure 9:**
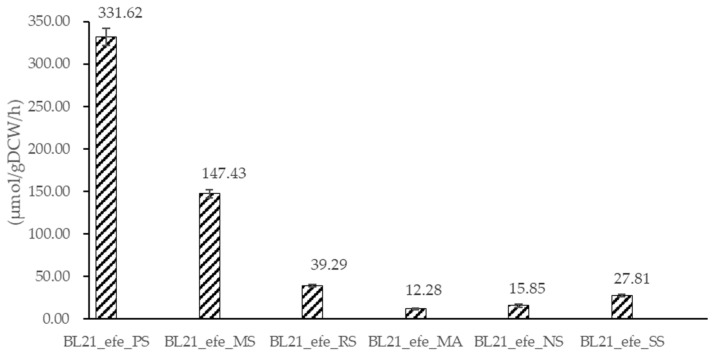
Ethylene production of BL21_*efe^+^* engineering strains in LB with 0.02% arabinose under 20 °C. Activity was calculated by the ethylene production per gram dry cell per hour.

Ethylene production results of the six Efes are shown in Figure 9. The activity of BL21_*efe_PS* is 331.61 μmol/gDCW/h. It is a little lower than the activity before using a *lac* promoter on a high-copy pUC18 vector (625.0 μmol/gDCW/h) or using a *tac* promoter on a medium-copy pBR322 vector (412.9 μmol/gDCW/h) [25]. Since plasmid pBAD in this study is a low-copy plasmid, it may lead to a lower RNA transcription and enzyme production in the cell. And it is reasonable that its activity in cells was slightly lower than in the previous study. For the other five strains, their ethylene production proves that all of them could work as an ethylene-forming enzyme, albeit at lower activity. Some of the factors contributing to that could be a lower protein expression or the formation of insoluble inclusion bodies that draft both amino acid building blocks and energy from the metabolism. Although Efe_MS and Efe_RS have relatively high activity in vitro at 20 °C, their transcript levels in engineered *E. coli* are low, as shown in qPCR results. It may be the primary reason for low enzymatic activity in vivo. Therefore, in the future, it may be worth using a high-copy plasmid to improve the RNA level of Efe_MS and Efe_RS to improve the activity in cells. The three enzymes of lowest activity—Efe_MA, Efe_NS, and Efe_SS are of cyanobacterial origin. Additionally, Efe_MS is from deltaproteobacteria, Efe_RS from betaproteobacteria, and Efe_PS from gammaproteobacteria, all much closer phylogenetically to *E. coli*. This could contribute to more efficient protein expression due to incompatibility of intracellular environment rare codons, amino acid sequence, challenging folding, etc. Besides, the changes in the β-strand of Efe_MS, Efe_MA, and Efe_RS may cause a lower activity ratio in cells to purified protein than Efe_MS and Efe_SS.

## 3. Discussion

The analysis of the molecular structure and sequence conservation (Figure 3, Table S2) shows that the sequences were conserved at the binding sites of Fe and AKG, while there were some non-conservative changes in the ARG binding sites and β-strands structures. But the change was not significant and not located in the key function sites. Combined with the successful production of ethylene in vitro and in vivo, these changes may have little effect on their work as Efe.

Experiments based on BL21_*efe_PS^+^* for optimising the induction environment (Figure 4) showed that the *efe* gene in pBAD plasmid was transcripted and expressed better at 20 °C than 30 °C and can work well in low concentration (0.02%) of arabinose as the inducer. This means that the cost of using arabinose as an inducer in large-scale production can be considerably low. Compared to the research of Digiacomo et al. [34] using the combination of arabinose and light-induced promoter at 37 °C with 5 mM (~0.075%) arabinose (~25 nmol/OD_600_/mL for 4 h), our ethylene production of BL21_*efe_PS^+^* (~3 μmol/OD_600_/mL for 5 h) was markedly improved at 20 °C with 0.02% arabinose. Low temperature avoided the mass formation of inclusion bodies, and an appropriate arabinose concentration enabled the gene to achieve better expression. Meanwhile, the volatile character of the product is not affected by the lower expression temperature. The analysis of enzyme activities also shows that Efes maintain stability at 20 °C for a long time (Figure 7). Therefore, incubating the expression cells at 20 °C with 0.02% arabinose was optimal for producing ethylene using this expression system. While SDS-PAGE analysis of the insoluble fraction confirmed the existence of inclusion bodies at 20 °C; their formation was reduced regarding other conditions. In the future, it would be worthwhile to utilize solubility partners to mitigate these deleterious effects, similar to the study of Ishihara et al. [24].

In our study, the enzyme activities of Efes in vitro were significantly higher than those in vivo (Table 2, Figure 9). However, due to the complexity of protein purification and the strict requirements of the reaction conditions for purified protein, producing ethylene in cells may be more suitable for large-scale production. This difference may prove that the addition of precursor substances plays an important role in increasing yield, which was also proved by Lynch et al. [35]: the addition of precursors (2 mM AKG and 3 mM ARG) can increase the yield in vivo by two to three times. Alternative metabolic methods to increase the flux towards AKG and ARG were also tested. With the analysis of the K_m_ of the six Efes, Efe_PS, Efe_MS, Efe_RS, Efe_NS have a higher level of K_m_ than the other two Efes. These four Efes may require lower addition of precursors in the medium and may be more suitable for industrial production. Combining the ethylene production in cells, the production of the five new Efes was 12.28–147.43 μmol/gDCW/h. It proves that all the strains can be successfully used in cells, but their activities are still lower than Efe_PS. Among the new Efes, Efe_MS showed the best ethylene production, and it may be a promising candidate for the industrial production of ethylene.

## 4. Materials and Methods

### 4.1. Efe Sequence Discovery

Potential ethylene forming enzymes sequences exhibiting 60–95% percent identity and 50–100% query coverage to the amino acid sequence of Efe of *P. syringae pv. phaseolicola* (protein ID: AAD16440.1) from cyanobacteria and proteobacteria (Table S1) were extracted from the NCBI database accessed on 1 February 2022 (https://www.ncbi.nlm.nih.gov/). They were selected by NCBI blastp, in the nt/nr database, excluding models (XM/XP) and uncultured/environmental sample sequences. MEGA-X software (Available at https://www.megasoftware.net/) [36] was accessed on 3 February 2022 to perform a bootstrap tree with the Maximum Likelihood method and JTT matrix-based model [37] for phylogenetic analysis after aligning sequences with ClustalW (gap opening: 10, gap extension: 0.2, delay divergent cutoff: 30%). The bootstrap consensus tree was inferred from 1000 replicates to represent the evolutionary relationship of the putative ethylene-forming proteins [38].

### 4.2. Sequence Analysis and Structural Modelling

Six Efe amino acid sequences from different strains were aligned and compared in Geneious Prime 1 January 2022 (Available at https://www.geneious.com/resources/#downloads). Structural models were built and accessed on 1 January 2022 by SWISS-MODEL (https://swissmodel.expasy.org/) and aligned in PyMOL (Available at https://pymol.org/2/) for secondary verification of the reliability of the structure, accessed on 1 January 2022. The structure of Efe in *P. syringae pv. phaseolicola* (PDB ID: 6vp4.1.A) was set as the reference for the aligning and modelling. In addition, the conservation of helix structures and ligand binding sites (AKG, ARG, iron-binding sites) were analysed.

### 4.3. Strains, Plasmids, and Culture Conditions

All plasmids were constructed in *E. coli* DH5α, and expression work was done in *E. coli* BL21. The expression plasmid pBAD vector) was used for protein production. The plasmid was a gift from Scott Gradia (Addgene plasmid # 37501; RRID: Addgene_37501). The plasmid contained *araBAD* arabinose promoter and ampicillin resistance gene.

*E. coli* was grown on LB medium or agar plates at 37 °C in an incubator (HZQ-X300C, Yiheng, Shanghai, China). Further, 50–100 μg/mL of ampicillin was added to maintain plasmids along with arabinose, which was used to induce the expression of *efe* genes. All the *E. coli* strains and plasmids used in this study are listed in Table 3.

After culturing overnight, cell culture was diluted into LB in a ratio of 1:50, cultured at 37 °C for 180 rpm until it grew to the concentration of OD_600_ = 0.4–0.6. Arabinose was added to the final concentration of 0.02%, the temperature was adjusted to 20 °C, and it was shaken for 5 h to express a protein or produce ethylene.

### 4.4. Construction of the Recombinant Plasmids

The protein sequences were obtained from NCBI, reverse translated, and synthesised by BGI Write (Beijing, China). The recombinant plasmids and primers used in this study are shown in Table 3 and Table 4. *Efe* genes were combined to EcoRV-predigested pBAD_LIC_cloning_vector using ClonExpressII One Step Cloning Kit (Vazyme, Nanjing) (Figure S3.). Primers for the assembly were designed with 15–20 bp overhangs (underlined). His-tag sequence (red) was added at the 3′-end of each gene to facilitate the protein purification step.

After the recombinant plasmids were constructed, they were transformed by the calcium ion transformation method initially to *E. coli* DH5α and ultimately to *E. coli* BL21.

Wherelowercase bases represent homology sequence, *ITALICS*—6 × His tag added to the C-terminal part of the protein.

### 4.5. Quantification of Cells and pH Detection of Culture

OD_600_ of the culture was tested for measuring cell density with an EPOCH microplate reader (BIOTEK, Winooski, VT, USA). The pH of the culture environment was measured with a PB-10 pH meter (SARTORIUS, Goettingen, Germany).

### 4.6. Real-Time RT-PCR Analysis

1.5 mL *E. coli* cells of each sample were collected by centrifugation at 4 °C at 8000 rpm for 3 min to isolate RNA with Total RNA kit I (Omega bio-tek, Norcross, GA, USA). PrimeScript™ RT reagent Kit with gDNA Eraser (Takara, Tokyo, Japan) was used to erase gDNA and reverse transcribe RNA to cDNA. qPCR was done with TB Green Premix Ex Taq II (TaKaRa) in a QuantStudio 5 real-time system (ABI, Thernofisher, Waltham, MA, USA). Primers for qPCR of the *efe* gene are F: 5’-ATGCCATAGCATTTTTATCC-3’ and R: 5′-GATTTAATCTGTATCAGG-3’. Standard curves were finished with 10-fold serial dilution of the standard fragments (around 100–200 bp, amplified by qPCR primers). They were used to ensure the PCR efficiency and calculate the RNA gene copies. A fragment of 16sRNA in *E. coli* was used as the reference gene for RT-qPCR. Its primers are F: 5’-ACTCCTACGGGAGGCAGCAG-3’ and R: 5’-ATTACCGCGGCTGCTGG-3’.

### 4.7. SDS-PAGE Analysis and Protein Purification

Cells were collected by centrifugation at 4 °C at 8000 rpm for 10 min. The pelleted fraction was suspended again in Binding buffer (20 mM Tris, 0.5 M NaCl, 5 mM imidazole, pH 7.8) or phosphate buffer (PBS). Cells were broken in ice using an Ultrasonic Cell Disruptor (SCIENTZ-IID, Ningbo, China). The supernatant and insoluble fraction were separately analysed with sodium dodecyl sulfate-polyacrylamide gel electrophoresis (SDS-PAGE). The proportion of target protein in it was calculated by ImageJ with its integrated density. A protein MS Q-E test (BGI, Guangzhou, China) was used to verify the expression results.

Efes were purified with Ni NTA Beads 6FF (Smart-life science, Shenzhen, China) according to the manufacturer’s instructions. They were washed and eluted with different concentrations of imidazole. Ultrafiltration centrifugal tubes (10 kDa, Sigma-Aldrich Merck, Darmstadat, Germany) were used to wash and concentrate the purified protein. The concentration of the purified protein was measured with BCA Protein Assay Kit (Thermo Scientific, Waltham, MA, USA).

### 4.8. Enzymatic Activity In Vitro

The activity of a purified protein in vitro was determined based on the conversion of AKG during the ethylene-forming reaction. The reaction system (800 μL) is consisted of 40 mM HEPES/NaOH (pH = 7.5), 0.2 mg/mL AKG, 0.5 mM ARG, 0.2 mM FeSO4, 1 mM L-histidine and 100 μL of 12 μg/mL purified proteins referring to the research of Katsuya [24]. The reaction was initiated by combining the enzyme and the reaction solution. Then it was incubated for 12 h in sterile 1.5 mL microcentrifuge tubes at constant temperatures and stirred (300 rpm). Subsequently, the samples were incubated for 5 min at 80 °C to inactivate the enzyme and cool down. AKG or ARG in the reaction system was set to different concentration gradients to analyse k_cat_ and K_m_ of the six Efes and was calculated in the following way: 100 μL of 100 μg/mL purified proteins were added in the reaction system and incubated for 20 min at 20 °C. The concentration of AKG and ARG were saturating, and their respective ranges were 0.021–0.685 mM and 0.005–0.50 mM.

The amount of AKG converted during the reaction was determined by HPLC 1260 Infinity (Agilent, USA) with Agilent Hi-Plex column and RID detector. AKG was diluted into different concentrations in ddH_2_O to make a standard curve. During the preparation of the reaction mixture, the same volume of ddH_2_O and protein solution from BL21_pBAD as the Efes solution was added to the reaction buffer as the negative control group. Enzyme activity was defined as the amount (1 μmol) of ethylene produced by 1 mg of enzyme per minute. The ethylene production activity and AKG conversion activity in cells were calculated as follows:

(1) $$\mathrm{Enzyme}\;\mathrm{activity}\;\left(\mathrm{Ethylene}\right)=2\mathrm{AKG}\;\mathrm{conversion}\;\mathrm{activity}\;=2\frac{{\Delta C}_{\mathrm{AKG}}}{C_{p}\cdot T}$$

ΔC_AKG_: AKG concentration difference in the system, measured by HPLC (μmol/mL); C_p_: concentration of the Efe protein, measured by BCA kit (mg/mL); T: reaction time, 12 h (h).

### 4.9. Ethylene Production in Cells

Ethylene production in the gas phase was analysed in a headspace of a 60 mL bottle with a 5 mL cell culture. Such setup provides enough co-substrate O_2_ and allows ethylene to be released into the headspace after formation in the cells. 1 mL air sample was injected to Agilent Technology 6850 GC FID with Porapak Q 3M × 1/8 column to measure the concentration of ethylene. Three biological replicates of each strain were prepared to measure ethylene production, while one was for density measurement with OD_600_. The dry cellular weight measurement was done after freeze-drying for 24 h with a FREEZOME2.5 Lyophilizer (Labconco, Kansas City, MO, USA). The activity of ethylene production in cells was calculated as follows:

(2) $$\mathrm{Ethylene}\;\mathrm{production}\;\mathrm{activity}=\frac{C\cdot \mathrm{Va}}{22.4\frac{\mu L}{\mu \mathrm{mol}}\cdot 1000\cdot T\cdot \mathrm{DCW}}$$

C: concentration of ethylene in the bottle, measured by GC (ppm); Va: gas volume in the bottle (mL); T: reaction time (h), 5 h; DCW: dry cellular weight, measured at the end of the reaction (g)

## 5. Conclusions

In this study, we identified and modelled five Efes selected by phylogenetic analysis and conservation of catalytic structure, heterologously expressed, and characterised them. The expression studies were performed in *E. coli*_BL21 using low-copy vector pBAD under the control of *araC* and *araBAD* promoter. The minor changes in structures were found in modelling and aligning, while all the five proteins retained the main framework of Efe. SDS-PAGE and qPCR results show that proteobacterial Efe_MS and Efe_RS had low mRNA levels, and some insoluble inclusion bodies were formed during the expression of these proteins. Improving the expression using a high-copy plasmid, changing the host, or improving the expression environment using fusion partners could facilitate higher ethylene yields. According to the comprehensive activity analysis in vitro and in vivo, the Efes have similar enzyme activity in vitro but very different enzyme activity in vivo. Nevertheless, activity in vitro and in vivo proves that all analysed enzymes exhibit ethylene-forming activity. They may be utilised in the clean production of ethylene and enzyme improvement, for example, using gene shuffling, which can be explored in the future. Comprehensive in vivo and in vitro enzyme activity for the five new Efes, Efe_MS has a lower Km of the precursor, AKG, and ARG, and the highest production in cells. It would be more appropriate as a promising candidate for the industrial production of ethylene.

## Figures and Tables

**Table 1:** Summary of fundamental kinetic parameters, K_m_, k_cat_, of six purified ethylene forming enzymes.

	K_m_ (ARG) (μM)	K_m_(AKG) (μM)	MM (kDa)	k_cat_ (ARG) (s^−1^)	k_cat_ (AKG) (s^−1^)
Efe_PS	22.4	25.5	40.27	0.814	0.793
Efe_MS	26.0	22.9	41.58	0.872	0.840
Efe_RS	15.1	19.8	40.27	0.913	0.893
Efe_MA	41.6	27.3	43.10	0.643	0.616
Efe_NS	21.5	24.9	42.33	0.723	0.695
Efe_SS	45.9	37.3	41.90	0.376	0.364

**Table 2:** Purification table for six purified ethylene forming enzymes, including specific and total enzyme activity. (U = 1 µmol of ethylene produced per minute), corresponding yield and enrichment.

Efes	Crude Extract		Purified Protein		Yield	Enrichment
	Total Enzyme Activity (U)	Specific Enzyme Activity (U/mg)	Total Enzyme Activity (U)	Specific Enzyme Activity (U/mg)		
Efe_PS	0.619 ± 0.052	0.011 ± 0.001	0.130 ± 0.005	0.536 ± 0.021	20.9%	48
Efe_MS	0.482 ± 0.040	0.008 ± 0.001	0.189 ± 0.008	0.566 ± 0.031	39.2%	70
Efe_RS	0.306 ± 0.023	0.007 ± 0.001	0.079 ± 0.004	0.630 ± 0.033	25.7%	87
Efe_MA	0.310 ± 0.029	0.005 ± 0.001	0.115 ± 0.008	0.378 ± 0.023	37.0%	74
Efe_NS	0.219 ± 0.017	0.006 ± 0.001	0.086 ± 0.004	0.464 ± 0.021	39.5%	79
Efe_SS	0.268 ± 0.002	0.006 ± 0.001	0.085 ± 0.006	0.205 ± 0.023	31.9%	36

**Table 3:** *E. coli* strains and plasmids used in this article.

Strains/Plasmids	Genotype	Source
***E. coli* strains**		
DH5α	F^−^ *φ80 lacZ ΔM15 Δ(lacZYA-argF)U169 endA1 recA1 hsdR17 (r_k_^−^, m_k_^+^) supE44 λ^−^ thi-1 gyrA96 relA1 phoA*	From ktsm-life
BL21(DE3)	F^−^ *ompT hsdS(r_B_^−^m_B_^−^) gal dcm*(DE3)	From ktsm-life
**Plasmids**		
Plasmid_#37501_pBAD_LIC_cloning_vector_(8A)	synthetic circular DNA with *pBR322* replication ori, *rop*, *bom*, *araC*, *araBAD* promoter, *Amp^r^*	Addgene_37501
PUC57-*efe_PS*	PUC57-*efe_PS* (from *P. syringae pv.* phaseolicola, Genbank: AF101058.1-AAD16440.1, optimised)	Synthesied by BGI Write
PUC57-*efe_MS*	PUC57-*efe_MS* (from *Myxococcus stipitatus* DSM 14675, NCBI Reference Sequence: WP_015351455.1, optimised)	Synthesied by BGI Write
PUC57-*efe_RS*	PUC57-*efe_RS* (from *Ralstonia solanacearum* strain IBSBF1503 plasmid, Genbank: WP_014618742.1, optimised)	Synthesied by BGI Write
PUC57-*efe_MA*	PUC57-*efe_MA* (from *Microcoleus asticus* IPMA8, GenBank: NQE34890, optimised)	Synthesied by BGI Write
PUC57-*efe_NS*	PUC57-*efe_NS* (from *Nostoc* sp. ATCC 43529, Genbank: RCJ18531.1, optimised)	Synthesied by BGI Write
PUC57-*efe_SS*	PUC57-*efe_SS* (from *Scytonema* sp. NIES-4073, NCBI Reference Sequence: WP_096562523.1, optimised)	Synthesied by BGI Write
pBAD-*efe_PS*	*pBR322* replication ori, *araC*, *araBAD* promoter, *efe_PS* with 6 × His-tag on the C-terminal, *Amp^r^*	This study
pBAD-*efe_MS*	*pBR322* replication ori, *araC*, *araBAD* promoter, *efe_MS* with 6 × His-tag on the C-terminal, *Amp^r^*	This study
pBAD-*efe_RS*	*pBR322* replication ori, *araC*, *araBAD* promoter, *efe_RS* with 6 × His-tag on the C-terminal, *Amp^r^*	This study
pBAD-*efe_MA*	*pBR322* replication ori, *araC*, *araBAD* promoter, *efe_MA* with 6 × His-tag on the C-terminal, *Amp^r^*	This study
pBAD-*efe*_*NS*	*pBR322* replication ori, *araC*, *araBAD* promoter, *efe_NS* with 6 × His-tag on the C-terminal, *Amp^r^*	This study
pBAD-*efe_SS*	*pBR322* replication ori, *araC*, *araBAD* promoter, *efe_SS* with 6 × His-tag on the C-terminal, *Amp^r^*	This study

**Table 4:** Primers for the construction of *E. coli* vectors expressing six ethylene forming enzymes were used in this study.

Genes	Orientation	Sequences of Oligonucleotides
*efe_PS*	F	actttaagaaggagatATAGATATGACTAACTTGCAAACCTTCGA
	R	tccttatggagttgggatCTA*ATGGTGATGGTGATGGTG*GGATCCTGTGGCTCGGG
*efe_MS*	F	actttaagaaggagatATAGATATGATTGAACTTGAGACCTTTCAACT
	R	tccttatggagttgggatCTA*ATGGTGATGGTGATGGTG*GCCAGCTGCGCGTTGCAG
*efe_RS*	F	actttaagaaggagatATAGATATGACAGGCCTTACCACATT
	R	tccttatggagttgggatCTA*ATGGTGATGGTGATGGTG*CGCAACCTCGCCCAGGG
*efe_MA*	F	actttaagaaggagatATAGATATGACTCATAAGTATCAAGAAAAGATCGA
	R	tccttatggagttgggatCTA*ATGGTGATGGTGATGGTG*CCGCTGTAGTTCAATGGCTT
*efe_NS*	F	actttaagaaggagatATAGATATGACCGATCTACAAACCTTTGAC
	R	tccttatggagttgggatCTA*ATGGTGATGGTGATGGTG*CAAATAGCTTGGCACTAAAGTCT
*efe_SS*	F	actttaagaaggagatATAGATATGACAGACCTGCAGACATTCC
	R	tccttatggagttgggatCTA*ATGGTGATGGTGATGGTG*GCGCTGGAGCGCATATG

## Data Availability

The original contributions presented in the study are included in the article/Supplementary Materials. Further inquiries can be directed to the corresponding author.

## Supplementary Materials

The following supporting information can be downloaded at: https://www.mdpi.com/article/10.3390/ijms23094500/s1.

## References

[1] Norton M. (2021). Polyethylene—The Material of Chance. Ten Materials That Shaped Our World.

[2] Easterbrook E.K., Allen R.D. (1987). Ethylene-Propylene Rubber. Rubber Technology.

[3] Bährle-Rapp M. (2007). Ethylene/Acrylic Acid Copolymer.

[4] Amin N.A. (2005). Production of gasoline range hydrocarbons from catalytic reaction of methane in the presence of ethylene over W/HZSM-5. Catal. Today.

[5] Jia-Xiang W. (1980). Development of ethylene production technology from petroleum hydrocarbon cracking. Petrochem. Ind..

[6] Chaogang X. (2000). Studies on catalytic pyrolysis process for ethylene production and its reaction mechanism. Pet. Process. Petrochem..

[7] Eckert C., Xu W., Xiong W., Lynch S., Ungerer J., Tao L., Gill R., Maness P.C., Yu J. (2014). Ethylene-forming enzyme and bioethylene production. Biotechnol. Biofuels.

[8] Morgan P.W., Drew M.C. (1997). Ethylene and plant responses to stress. Physiol. Plant..

[9] Hall M., Smith A.R. (1995). Ethylene and the responses of plants to stress. Bulg. J. Plant Physiol..

[10] Wang K.L.C., Li H., Ecker J.R. (2002). Ethylene Biosynthesis and Signaling Networks. Plant Cell.

[11] Weingart H., Ullrich H., Geider K., Völksch B. (2001). The Role of Ethylene Production in Virulence of Pseudomonas syringae pvs. glycinea and phaseolicola. Phytopathology.

[12] Shipston N., Bunch A. (1989). The Physiology of L-Methionine Catabolism to the Secondary Metabolite Ethylene by Escherichia coli. J. Gen. Microbiol..

[13] Mansouri S., Bunch A. (1989). Bacterial ethylene synthesis from 2-oxo-4-thiobutyric acid and from methionine. J. Gen. Microbiol..

[14] Yang S.F. (1967). Biosynthesis of ethylene: Ethylene formation from methional by horseradish peroxidase. Arch. Biochem. Biophys..

[15] Kazuhiro N., Ogawa T., Fujii T., Tazaki M., Tanase S., Morino Y., Fukuda H. (1991). Purification and properties of an ethylene-forming enzyme from Pseudomonas syringae pv. Phaseolicola PK2. J. Gen. Microbiol..

[16] Li M., Ho P.Y., Yao S., Shimizu K. (2006). Effect of sucA or sucC gene knockout on the metabolism in Escherichia coli based on gene expressions, enzyme activities, intracellular metabolite concentrations and metabolic fluxes by 13C-labeling experiments. Biochem. Eng. J..

[17] Xiao D., Zeng L., Yao K., Kong X., Wu G., Yin Y. (2016). The glutamine-alpha-ketoglutarate (AKG) metabolism and its nutritional implications. Amino Acids.

[18] Herr C.Q., Hausinger R.P. (2018). Amazing Diversity in Biochemical Roles of Fe(II)/2-Oxoglutarate Oxygenases. Trends Biochem. Sci..

[19] Fukuda H., Ogawa T., Tazaki M., Nagahama K., Fujii T., Tanase S., Morino Y. (1992). Two reactions are simultaneously catalysed by a single enzyme: The arginine-dependent simultaneous formation of two products, ethylene and succinate, from 2-oxoglutarate by an enzyme from Pseudomonas syringae. Biochem. Biophys. Res. Commun..

[20] Martinez S., Fellner M., Herr C.Q., Ritchie A., Hu J., Hausinger R.P. (2017). Structures and Mechanisms of the Non-Heme Fe(II)- and 2-Oxoglutarate-Dependent Ethylene-Forming Enzyme: Substrate Binding Creates a Twist. J. Am. Chem. Soc..

[21] Li M., Martinez S., Hausinger R.P., Emerson J.P. (2018). Thermodynamics of Iron(II) and Substrate Binding to the Ethylene-Forming Enzyme. Biochemistry.

[22] Freebairn H.T., Buddenhagen I.W. (1964). Ethylene Production by Pseudomonas solanacearum. Nature.

[23] Fukuda H., Ogawa T., Ishihara K., Fujii T., Nagahama K., Omata T., Inoue Y., Tanase S., Morino Y. (1992). Molecular cloning in Escherichia coli, expression, and nucleotide sequence of the gene for the ethylene-forming enzyme of Pseudomonas syringae pv. phaseolicola PK2. Biochem. Biophys. Res. Commun..

[24] Ishihara K., Matsuoka M., Inoue Y., Tanase S., Ogawa T., Fukuda H. (1995). Overexpression and In Vitro Reconstitution of the Ethylene-Forming Enzyme from Pseudomonas syringae. J. Ferment. Bioeng..

[25] Ishihara K., Matsuoka M., Ogawa T., Fukuda H. (1996). Ethylene production using a broad-host-range plasmid in Pseudomonas syringae and Pseudomonas putida. J. Ferment. Bioeng..

[26] Pirkov I., Albers E., Norbeck J., Larsson C. (2008). Ethylene production by metabolic engineering of the yeast Saccharomyces cerevisiae. Metab. Eng..

[27] Chen X., Liang Y., Hua J., Tao L., Qin W., Chen S. (2010). Overexpression of bacterial ethylene-forming enzyme gene in Trichoderma reesei enhanced the production of ethylene. Int. J. Biol. Sci..

[28] Sakai M., Ogawa T., Matsuoka M., Fukuda H. (1997). Photosynthetic conversion of carbon dioxide to ethylene by the recombinant cyanobacterium, Synechococcus sp. PCC 7942, which harbors a gene for the ethylene-forming enzyme of Pseudomonas syringae. J. Ferment. Bioeng..

[29] Takahama K., Matsuoka M., Kazuhiro N., Ogawa T. (2003). Construction and Analysis of a Recombinant Cyanobacterium Expressing a Chromosomally Inserted Gene for an Ethylene-Forming Enzyme at the psbAI Locus. J. Biosci. Bioeng..

[30] Ungerer J., Tao L., Davis M., Ghirardi M., Maness P.-C., Yu J. (2012). Sustained Photosynthetic Conversion of Atmospheric CO2 to Ethylene in Recombinant Cyanobacterium Synechocystis 6803. Environ. Sci. Technol..

[31] Tao L., Dong H.-J., Chen X., Chen S.-F., Wang T.-H. (2008). Expression of ethylene-forming enzyme (EFE) of Pseudomonas syringae pv. glycinea in Trichoderma viride. Appl. Microbiol. Biotechnol..

[32] Wang J.-P., Wu L.-X., Xu F., Lv J., Jin H.-J., Chen S.-F. (2010). Metabolic engineering for ethylene production by inserting the ethylene-forming enzyme gene (efe) at the 16S rDNA sites of Pseudomonas putida KT2440. Bioresour. Technol..

[33] Guerrero F., Carbonell V., Cossu M., Correddu D., Jones P.R. (2012). Ethylene synthesis and regulated expression of recombinant protein in Synechocystis sp. PCC 6803. PLoS ONE.

[34] Digiacomo F., Girelli G., Aor B., Marchioretti C., Pedrotti M., Perli T., Tonon E., Valentini V., Avi D., Ferrentino G. (2014). Ethylene-Producing Bacteria That Ripen Fruit. ACS Synth. Biol..

[35] Lynch S., Eckert C., Yu J., Gill R., Maness P.C. (2016). Overcoming substrate limitations for improved production of ethylene in E. coli. Biotechnol. Biofuels.

[36] Kumar S., Stecher G., Li M., Knyaz C., Tamura K. (2018). MEGA X: Molecular Evolutionary Genetics Analysis across Computing Platforms. Mol. Biol. Evol..

[37] Jones D.T., Taylor W.R., Thornton J.M. (1992). The rapid generation of mutation data matrices from protein sequences. Comput. Appl. Biosci..

[38] Felsenstein J. (1985). Confidence limits on phylogenies: An approach using the bootstrap. Evolution.

