# Supplementary material for: Discovery of Five New Ethylene-Forming Enzymes for Clean Production of Ethylene in E. coli
Source: Int J Mol Sci. 2022 Apr 19;23(9):4500. doi: 10.3390/ijms23094500 (PMC9101411; doi:10.3390/ijms23094500)
Supplement: Supplementary file 1 [file ijms-23-04500-s001.zip › ijms-1661780-supplementary.pdf]

Table S1. Sequences for phylogenetic analysis.

Names	Accession	Description	Organisms	Length [aa]	Taxonomy
AF101058-AAD16440.1	AAD16440.1	ethylene-forming enzyme	*Pseudomonas syringae pv. Phaseolicola*	350	Bacteria; Proteobacteria; Gammaproteobacteria; Pseudomonadales; Pseudomonadaceae; *Pseudomonas*
MBW4490662	MBW4490662.1	MAG: hypothetical protein KME12_23050	*Trichocoleus desertorum* ATA4-8-CV12	339	Bacteria; Cyanobacteria; Synechococcales; Trichocoleusaceae; *Trichocoleus*
MBW4538205	MBW4538205.1	MAG: hypothetical protein KME43_03555	*Myxacorys chilensis* ATA2-1-KO14	352	Bacteria; Cyanobacteria; Pseudanabaenales; Leptolyngbyaceae; *Myxacorys*; *Myxacorys chilensis*
RAM52958	RAM52958.1	MAG: MFS transporter	*Hapalosiphonaceae cyanobacterium* JJU2	360	Bacteria; Cyanobacteria; Nostocales; Hapalosiphonaceae
RCJ18531	RCJ18531.1	MFS transporter	Nostoc sp. ATCC 43529	361	Bacteria; Cyanobacteria; Nostocales; Nostocaceae; *Nostoc*
RML49514	RML49514.1	2OG-Fe oxygenase	*Pseudomonas syringae pv. pisi*	344	Bacteria; Proteobacteria; Gammaproteobacteria; Pseudomonadales; Pseudomonadaceae; *Pseudomonas*; *Pseudomonas syringae*
TMH32900	TMH32900.1	MAG: isopenicillin N synthase family oxygenase	*Betaproteobacteria bacterium*	352	Bacteria; Proteobacteria; Betaproteobacteria
WP_014618742	WP_014618742.1	isopenicillin N synthase family oxygenase	*Ralstonia solanacearum*	347	Bacteria; Proteobacteria; Betaproteobacteria; Burkholderiales; Burkholderiaceae; *Ralstonia*
WP_015351455	WP_015351455.1	isopenicillin N synthase family oxygenase	*Myxococcus stipitatus*	360	Bacteria; Proteobacteria; Deltaproteobacteria; Myxococcales; Cystobacterineae; Myxococcaceae; *Myxococcus*
WP_019362686	WP_019362686.1	isopenicillin N synthase family oxygenase	*Pseudomonas fuscovaginae*	350	Bacteria; Proteobacteria; Gammaproteobacteria; Pseudomonadales; Pseudomonadaceae; *Pseudomonas*
WP_057426137	WP_057426137.1	isopenicillin N synthase family oxygenase	*Pseudomonas syringae*	345	Bacteria; Proteobacteria; Gammaproteobacteria; Pseudomonadales; Pseudomonadaceae; *Pseudomonas*
WP_063343083	WP_063343083.1	MULTISPECIES: isopenicillin N synthase family oxygenase	*Pseudomonas*	353	Bacteria; Proteobacteria; Gammaproteobacteria; Pseudomonadales; Pseudomonadaceae

WP_071780333	WP_071780333.1	isopenicillin N synthase family oxygenase	*Fischerella sp.* PCC 9431	385	Bacteria; Cyanobacteria; Nostocales; Hapalosiphonaceae; *Fischerella*
WP_074950276	WP_074950276.1	isopenicillin N synthase family oxygenase	*Myxococcus fulvus*	360	Bacteria; Proteobacteria; Deltaproteobacteria; Myxococcales; Cystobacterineae; Myxococcaceae; *Myxococcus*
WP_096562523	WP_096562523.1	isopenicillin N synthase family oxygenase	*Scytonema sp.* NIES-4073	359	Bacteria; Cyanobacteria; Nostocales; Scytonemataceae; *Scytonema*; unclassified *Scytonema*
WP_116665714	WP_116665714.1	isopenicillin N synthase family oxygenase	*Pseudomonas sp.* GL93	346	Bacteria; Proteobacteria; Gammaproteobacteria; Pseudomonadales; Pseudomonadaceae; *Pseudomonas*
WP_123496614	WP_123496614.1	isopenicillin N synthase family oxygenase	*Pseudomonas frederiksbergensis*	344	Bacteria; Proteobacteria; Gammaproteobacteria; Pseudomonadales; Pseudomonadaceae; *Pseudomonas*
WP_131118892	WP_131118892.1	isopenicillin N synthase family oxygenase	*Mastigocladus laminosus*	360	Bacteria; Cyanobacteria; Nostocales; Hapalosiphonaceae; *Mastigocladus*
WP_141641058	WP_141641058.1	isopenicillin N synthase family oxygenase	*Myxococcus*	362	Bacteria; Proteobacteria; Deltaproteobacteria; Myxococcales; Cystobacterineae; Myxococcaceae; *Myxococcus*
WP_163861341	WP_163861341.1	isopenicillin N synthase family oxygenase	*Myxococcus eversor*	360	Bacteria; Proteobacteria; Deltaproteobacteria; Myxococcales; Cystobacterineae; Myxococcaceae; *Myxococcus*
WP_163989172	WP_163989172.1	isopenicillin N synthase family oxygenase	*Pyxidicoccus caerfyrddinensis*	358	Bacteria; Proteobacteria; Deltaproteobacteria; Myxococcales; Cystobacterineae; Myxococcaceae; *Pyxidicoccus*
WP_169343509	WP_169343509.1	isopenicillin N synthase family oxygenase	*Pyxidicoccus fallax*	356	Bacteria; Proteobacteria; Deltaproteobacteria; Myxococcales; Cystobacterineae; Myxococcaceae; *Pyxidicoccus*
WP_174475597	WP_174475597.1	isopenicillin N synthase family oxygenase	*Myxococcus sp.* CA033	360	Bacteria; Proteobacteria; Deltaproteobacteria; Myxococcales; Cystobacterineae; Myxococcaceae; *Myxococcus*; unclassified *Myxococcus*
WP_176434236	WP_176434236.1	isopenicillin N synthase family oxygenase	*Myxococcus sp.* AM011	360	Bacteria; Proteobacteria; Deltaproteobacteria; Myxococcales; Cystobacterineae; Myxococcaceae; *Myxococcus*; unclassified *Myxococcus*
WP_181080955	WP_181080955.1	isopenicillin N synthase family oxygenase	*Pseudomonas lurida*	351	Bacteria; Proteobacteria; Gammaproteobacteria; Pseudomonadales; Pseudomonadaceae; *Pseudomonas*

WP_181286820	WP_181286820.1	isopenicillin N synthase family oxygenase	*Pseudomonas brassicacearum*	346	Bacteria; Proteobacteria; Gammaproteobacteria; Pseudomonadales; Pseudomonadaceae; *Pseudomonas*
WP_191133867	WP_191133867.1	isopenicillin N synthase family oxygenase	*Phormidium tenue*	352	Bacteria; Cyanobacteria; Oscillatoriophycideae; Oscillatoriales; Oscillatoriaceae; *Phormidium*
WP_193874723	WP_193874723.1	isopenicillin N synthase family oxygenase	*Oculatella sp.* LEGE 06141	356	Bacteria; Cyanobacteria; Pseudanabaenales; Oculatellaceae; *Oculatella*; unclassified *Oculatella*
WP_206715722	WP_206715722.1	isopenicillin N synthase family oxygenase	*Myxococcus* sp. SCHIC003	360	Bacteria; Proteobacteria; Deltaproteobacteria; Myxococcales; Cystobacterineae; Myxococcaceae; *Myxococcus;* unclassified *Myxococcus*
WP_216670419	WP_216670419.1	hypothetical protein	*Microcoleus asticus*	369	Bacteria; Cyanobacteria; Oscillatoriophycideae; Oscillatoriales; Microcoleaceae; *Microcoleus*
WP_217855079	WP_217855079.1	MFS transporter	*Pseudomonas asgharzadehiana*	339	Bacteria; Proteobacteria; Gammaproteobacteria; Pseudomonadales; Pseudomonadaceae; *Pseudomonas*
WP_223018911	WP_223018911.1	MFS transporter	*Pseudomonas sp*. P867	346	Bacteria; Proteobacteria; Gammaproteobacteria; Pseudomonadales; Pseudomonadaceae; *Pseudomonas*
WP_223745345	WP_223745345.1	MFS transporter	*Myxococcus sp*. XM-1-1-1	360	Bacteria; Proteobacteria; Deltaproteobacteria; Myxococcales; Cystobacterineae; Myxococcaceae; *Myxococcus;* unclassified *Myxococcus*
WP_223781934	WP_223781934.1	MFS transporter	*Myxococcus sp*. AS-1-15	352	Bacteria; Proteobacteria; Deltaproteobacteria; Myxococcales; Cystobacterineae; Myxococcaceae; *Myxococcus;* unclassified *Myxococcus*
WP_229496330	WP_229496330.1	hypothetical protein	*Nostoc mirabile*	360	Bacteria; Cyanobacteria; Nostocales; Nostocaceae; *Nostoc*

Table S2. Parameters of the five constructed models based on 6VP4.1.A.

**Proteins**	**Oligo-State**	**Seq Identity**	**GMQE**	**QMEANDisCo Global**	**Ligands**
Efe_MS	Monomer	63.29%	0.87	0.86 ± 0.05	1xAKG, 1xFE
Efe_RS	Monomer	61.40%	0.87	0.84 ± 0.05	1xAKG, 1xFE
Efe_MA	Monomer	67.74%	0.85	0.87 ± 0.05	1xARG, 1xAKG, 1xFE
Efe_NS	Monomer	67.25%	0.87	0.87 ± 0.05	1xAKG, 1xFE
Efe_SS	Monomer	68.10%	0.88	0.87 ± 0.05	1xARG, 1xAKG, 1xFE

* 1 x AKG: α-ketoglutaric acid ligand binding sites; 1 x ARG: arginine ligand binding sites; 1 x FE: FE (III) ion ligand binding sites.

*GMQE (Global Model Quality Estimate) and QMEANDisCo global (QMEAN: Qualitative Model Energy Analysis) give an overall model quality measurement between 0 and 1, with higher numbers indicating higher l expected quality. GMQE is coverage dependent, i.e., a model covering only half of the target sequence is unlikely to get a score above 0.5 QMEANDisCo on the other hand evaluates the model 'as is' without explicit coverage dependency.

(a)

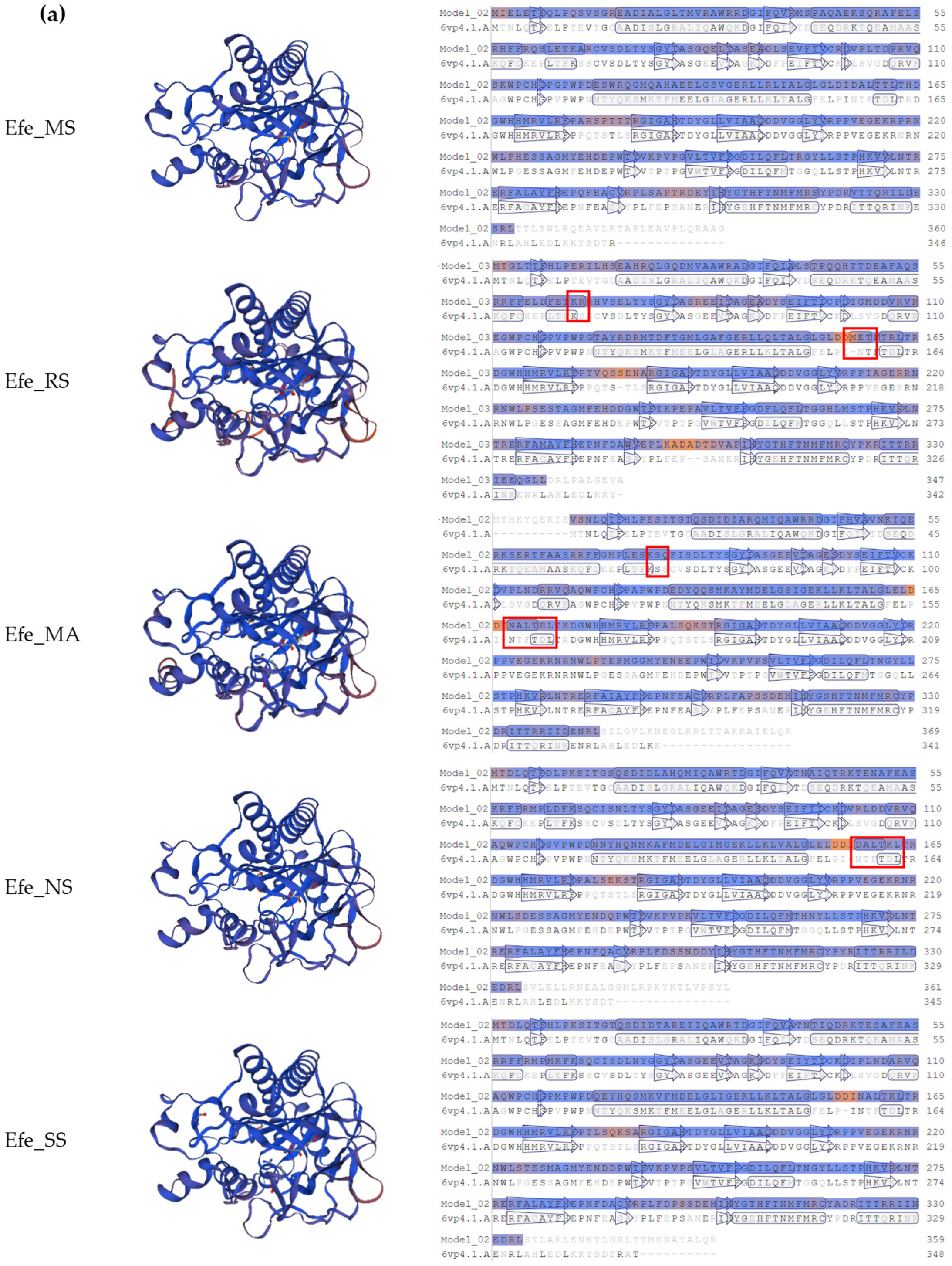

(b)

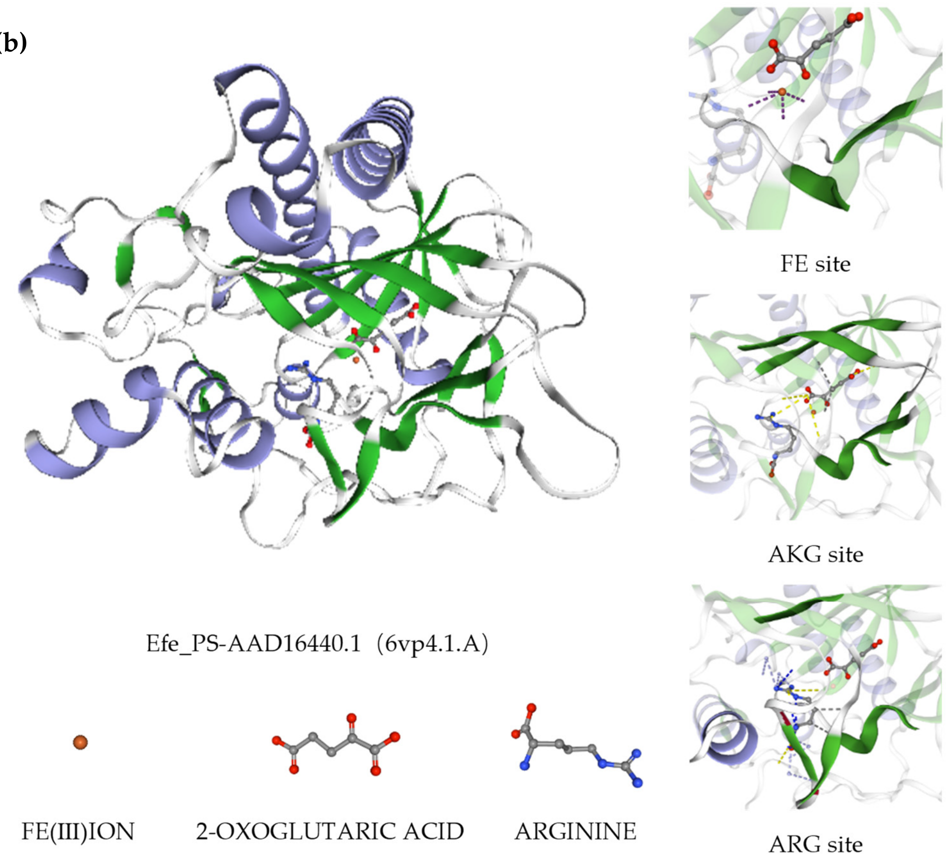

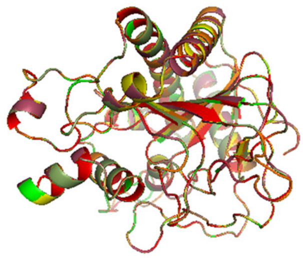

All the six

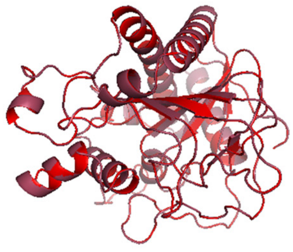

Efe_MS RMSD=0.057 (322 to 322 atoms)

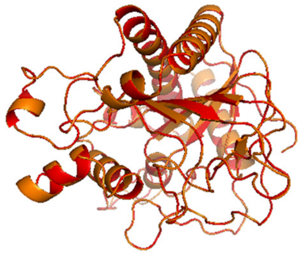

Efe_RS RMSD=0.056 (297 to 297 atoms)

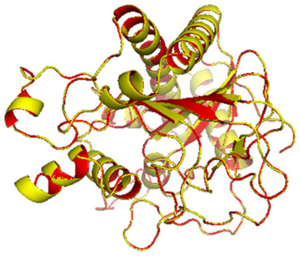

Efe_MA RMSD=0.055 (312 to 312 atoms)

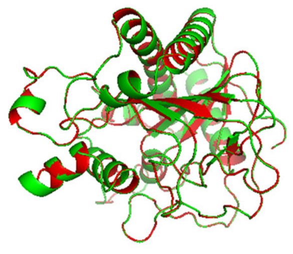

Efe_NS RMSD=0.054 (314 to 314 atoms)

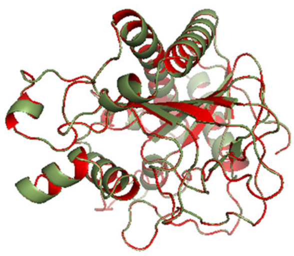

Efe_SS RMSD=0.058 (318 to 318 atoms)

Figure S1. Model of Efes, 6VP4.1.A and its active sites. (**a**) Efe models constructed by SWISS MODEL and structure aligning to 6VP4.1.A. They are shown by confidence level: blue—red: high confidence—low confidence. α-helix: labeled with the arrow; β-helix: labeled with square. Little change shows in α-helix or β-helix and most sites are in high confidence. Changes are marked by red box. (**b**) Model of 6VP4.1.A and its active sites (α-helix: purple; β-helix: green; active sites are darker in the right figures). (c) Structure alignment by PyMOL between five Efes and 6vp4.1.A (in red). RMSD root-mean-square deviation, a parameter used to indicate differences in atomic positions between protein structures. RMSDs of these five aligning are all less than 0.1 (abnormal atoms were rejected during the cycle), which means these models have little difference in structures. There are slight fluctuations in atomic positions between protein structures, while the main frameworks remain the same.

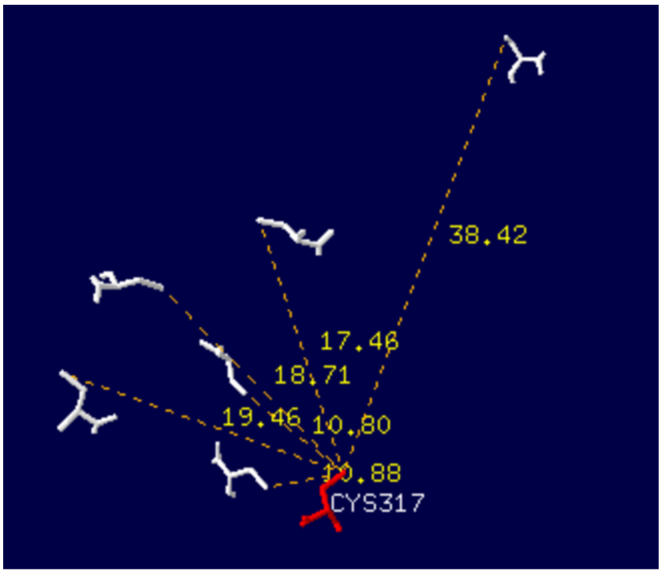

Figure S2. Analysis of disulfide bond formation of cysteine residue in the original model 6VP4.1.A.

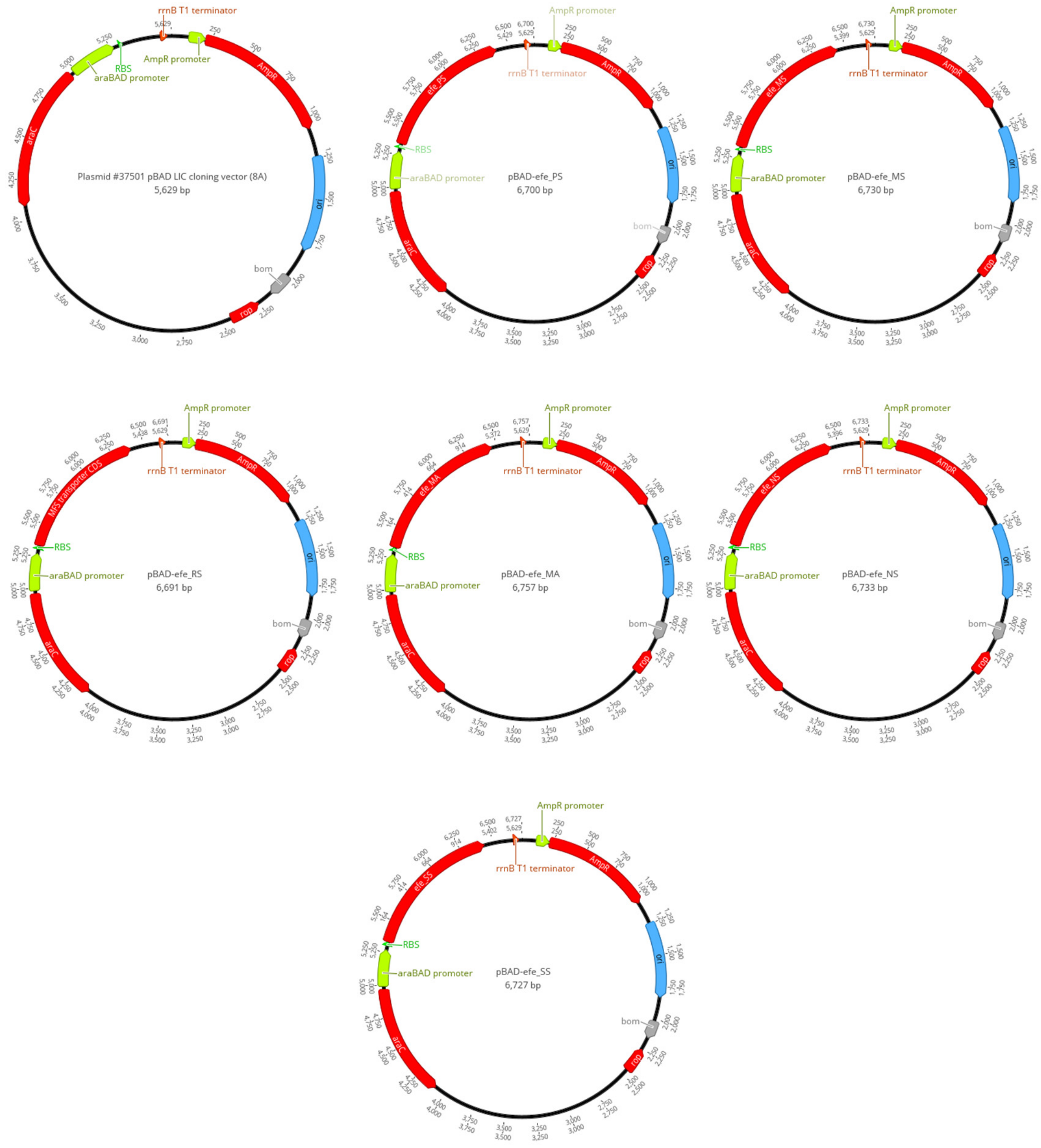

Figure S3. Plasmids profiles of pBAD_LIC_cloning vector and the six recombinant plasmids.

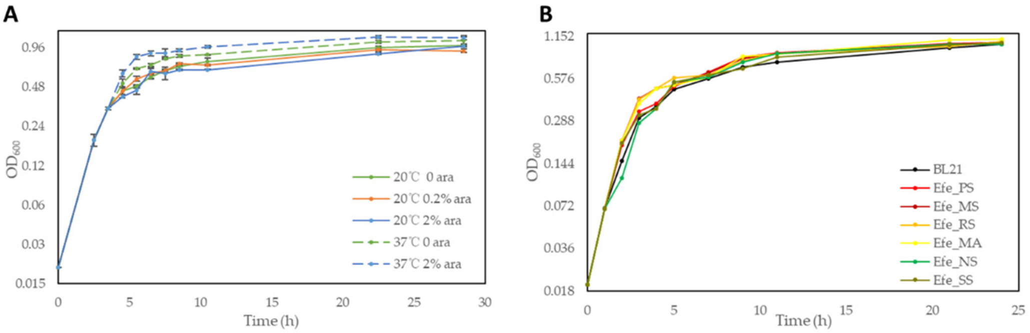

Figure S4. Growth curves of BL21_*efe*+ in LB medium with arabinose and antibiotic (ampicillin) in low temperature. (**A**). Growth in LB with arabinose concentration of 0 (green), 0.2% (orange) and 2% (blue) under 20 °C (full line) or 37 °C (dashed line); (**B**). Growth curve of the six engineering strains in LB with 2% arabinose and 50 μg/mL ampicillin: black: BL21_WT; red: Efe_PS; rose red: Efe_MS; orange: Efe_RS; yellow: Efe_MA; green: Efe_NS; brown: Efe_SS.

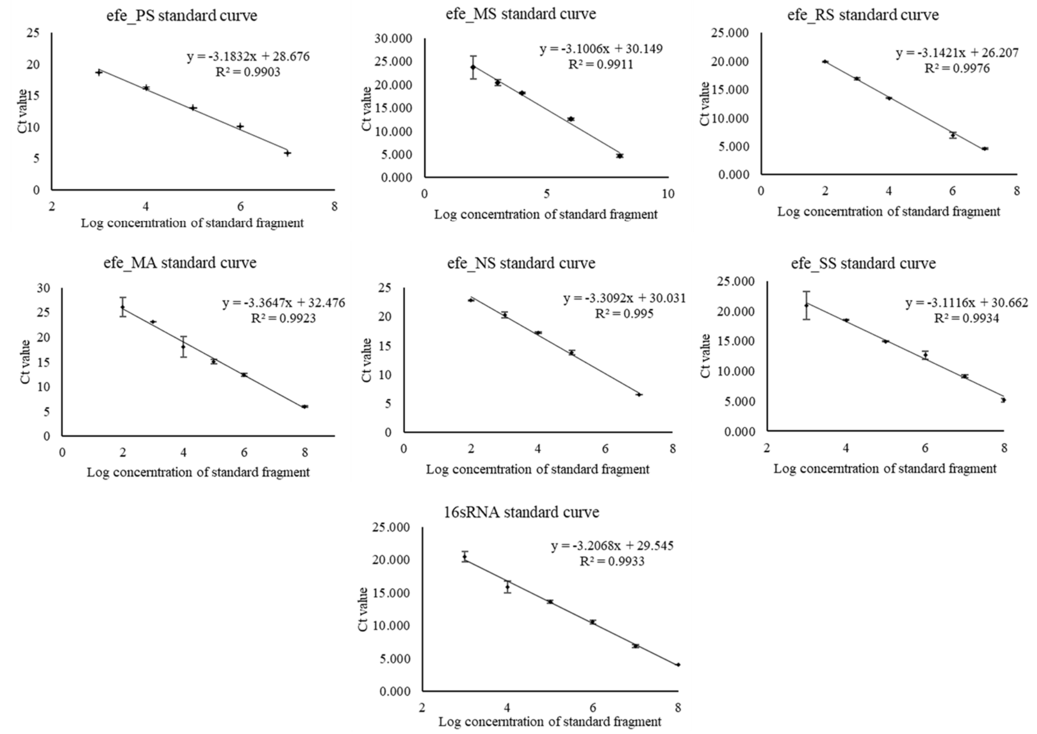

Figure S5. RT-qPCR standard curves of *efe* genes and 16sRNA gene.

Table S3. Protein MS (Q-E test) results of Efes.

Accession	Mass	Score	Matches1	Sequences2	emPAI3	Coverage
Efe_NS	39593	9040	268(233)	35(27)	49.86	71%
Efe_PS	39819	3179	160(113)	37(20)	12.90	70%
Efe_RS	41756	2542	89(70)	30(18)	12.35	68%
Efe_MS	40953	2060	129(74)	32(15)	8.50	58%
Efe_SS	39223	1406	70(33)	24(10)	1.44	47%
Efe_MA	42477	634	53(24)	21(10)	1.85	30%

[1] Matches: the total number of peptide matches, in parentheses, is the number of matches above the significance threshold; [2]Sequences: the total number of matched sequences is the number of sequences higher than the significance threshold in parentheses; [3]emPAI: $10^{(Nobserved/Nobservable)-1}$

Efe_PS Score: 3179

```
  1 MTNLQTFELP TEVTGCAADI SLGRALIQAW QKDGIFQIKT DSEQDRKTQE
 51 AMAASKQFCK EPLTFKSSCV SDLTYSGYVA SGEEVTAGKP DFPEIFTVCK
101 DLSVGDQRVK AGWPCHGPVP WPNNTYQKSM KTFMEELGLA GERLLKLTAL
151 GFELPINTFT DLTRDGWHHM RVLRFPPQTS TLSRGIGAHT DYGLLVIAAQ
201 DDVGGLYIRP PVEGEKRNRN WLPGESSAGM FEHDEPWTFV TPTPGVWTVF
251 PGDILQFMTG GQLLSTPHKV KLNTRERFAC AYFHEPNFEA SAYPLFEPSA
301 NERIHYGEHF TNMFMRCYPD RITTQRINKE NRLAHLEDLK KYSDTRATGS
351 _
```

Efe_MS Score: 2060

```
  1 MIELETFQLP QSVSGREADI ALGLTMVRAW RRDGIFQVRM SPAQAEKSQR
 51 AFELSRHFFR QSLETKARCV SDLTYSGYIA SGQELTASEA DLSEVFTVCR
101 DVPLTDPRVQ SKWPCHGPGP WPDESWRQGM QAHAEELGSV GERLLRLIAL
151 GLGLDIDALT TLTHDGWHHM RVLRFPARSP TTTRGIGAHT DYGLLVIAAQ
201 DDVGGLYVRP PVEGEKRPRN WLPHESSAGM YEHDEPWTYV KPVPGVLTVF
251 PGDILQFLTR GYLLSTPHKV VLNTRERFAL AYFHEPQFEA CVRPLSAPTR
301 DEYIHYGTHF TNMFMRSYPD RVTTQRILDE SRLTTLSWLR QEAVLRTAPL
351 EAVPLQRAAG _
```

Efe_NS Score: 9040

```
  1 MTGLTTFHLP ERILHSEAHR QLGQDMVAAW RADGIFQIAL STPQQHTTDE
 51 AFAQSRRFFE LDFETKRRHV SELTYSGYIA SREEITAGEA DYSEIFTICP
101 DIGMDDVRVR EGWPCHGPVP WPGTAYRDRM TDFTGMLGAF GERLLQLTAL
151 GLGLDDMETF TRLTRDGWHH MRVLRFPTVQ SSENARGIGA HTDYGLLVIA
201 AQDDVGGLYV RPPIAGERRN RNWLPSESTA GMFEHDDGWT FIKPEPAVLT
251 VFPGDFLQFL TGGHLMSTPH KVRLNTRERF AMAYFHEPNF DAWVEPLKAD
301 ADTDVAPIHY GTHFTNMFMR CYPKRITTRR IEEQGLLDRL PALGEVA_
```

Efe_MA Score: 634

```
  1 MTHKYQEKIE VSNLQIFHLP ESITGIQSDI DIARQMIQAW RRDGIFHVAV
 51 NKIQERKSER TFAASRRFFG MPLESKSQFI SDLTYSGYIA SGEEVTAGES
101 DYSEIFTVCK DVPLNDRRVQ AQWPCHGPAP WPDEDYQQSM KAYMDELGSI
151 GEKLLKLTAL GLELDDINAL TELTKDGWHH MRVLRFPALS QKSTRGIGAH
201 TDYGLLVIAA QDDVGGLYIR PPVEGEKRNR NWLPTESMGG MYENEEPWIL
251 VKPVPSVLTV FPGDILQFLT NGYLLSTPHK VRLNTRERFA IAYFHEPNFE
301 ACVRPLFAPS SDEHIHYGSH FTNMFMRCYP DRITTRRIID ENRLSILGVL
351 KNEGLRRLTT AKKAIELQR
```

Efe_RS Score: 2542

```
  1 MTDLQTFDLP KSITGSQSDI DLAHQMIQAW RTDGIFQVAT NAIQTRKTEN
 51 AFEASKRFFR MPLDFKSQCI SNLTYSGYIA SGEEITAGES DYSEIFTICK
101 DVRLDDVRVQ AQWPCHGSVP WPDNNYHQNM KAFMDELGIM GEKLLKLVAL
151 GLELDDIDAL TKLTRDGWHH MRVLRFPALS EKSTRGIGAH TDYGLLVIAA
201 QDDVGGLYVR PPVEGEKRNR NWLSDESSAG MYENDQPWTF VKPVPKVLTV
251 FPGDILQFMT HNYLLSTPHK VRLNTRERFA LAYFHEPNFQ ACVRPLFDSS
301 NDDYIHYGTH FTNMFMRCYP YRITTRRILD EDRLSVLELL RNEALGGMLR
351 PKYKTLVPSY L
```

Efe_SS Score: 1406

```
  1 MTDLQTFHLP KSITGTQSDI DTAREIIQAW RTDGIFQVAT NTIQDRKTES
 51 AFEASRRFFR MPMKFKSQCI SDLNYGGYIA SGEEVTAGKS DYSEIYTICK
101 DIPLNDARVQ AQWPCHGPMP WPDQEYHQSM KVFMDELGLI GEKLLKLTAL
151 GLGLDDINAL TKLTRDGWHH MRVLRFPTLS QKSARGIGAH TDYGLLVIAA
201 QDDVGGLYIR PPVEGEKRNR NWLSTESMAG MYENDDPWTF VKPVPSVLTV
251 FPGDILQFLT NGYLLSTPHK VRLNTRERFA LAYFHEPNFD ACVRPLFDPS
301 SDEHIHYGTH FTNMFMRCYA DRITTRRIIN EDRLSILARL ENKTLGRLTT
351 MKNAYALQR
```

Figure S6. Protein MS sequence alignment.
